# Application of Cerium (IV) as an Oxidimetric Agent for the Determination of Ethionamide in Pharmaceutical Formulations

**DOI:** 10.1155/2016/5410573

**Published:** 2016-10-13

**Authors:** Kanakapura Basavaiah, Nagib A. S. Qarah, Sameer A. M. Abdulrahman

**Affiliations:** ^1^Department of Chemistry, University of Mysore, Manasagangotri, Mysore 570 006, India; ^2^Department of Chemistry, Faculty of Education and Sciences Rada'a, Al-Baydha University, Al Bayda, Yemen

## Abstract

Two simple methods are described for the determination of ethionamide (ETM) in bulk drug and tablets using cerium (IV) sulphate as the oxidimetric agent. In both methods, the sample solution is treated with a measured excess of cerium (IV) solution in H_2_SO_4_ medium, and after a fixed standing time, the residual oxidant is determined either by back titration with standard iron (II) solution to a ferroin end point in titrimetry or by reacting with o-dianisidine followed by measurement of the absorbance of the orange-red coloured product at 470 nm in spectrophotometry. In titrimetry, the reaction proceeded with a stoichiometry of 1 : 2 (ETM : Ce (IV)) and the amount of cerium (IV) consumed by ETM was related to the latter's amount, and the method was applicable over 1.0–8.0 mg of drug. In spectrophotometry, Beer's law was obeyed over the concentration range of 0.5–5.0 *μ*g/mL ETM with a molar absorptivity value of 2.66 × 10^4^ L/(mol·cm). The limits of detection (LOD) and quantification (LOQ) calculated according to ICH guidelines were 0.013 and 0.043 *μ*g/mL, respectively. The proposed titrimetric and spectrophotometric methods were found to yield reliable results when applied to bulk drug and tablets analysis, and hence they can be applied in quality control laboratories.

## 1. Introduction

Ethionamide (ETM), chemically known as 2-ethylthioisonicotinamide, is a second-line orally administered drug that is used for the treatment of multidrug resistant tuberculosis [1]. The drug has been in use since 1960s, because it is cheap, easily available, relatively nontoxic, and efficacious [2]. ETM is a structural analog of isoniazid [3, 4] and is found to inhibit mycolic acid biosynthesis [5] with good bioavailability [6].

The drug is official in the British Pharmacopoeia [7], which describes a titrimetric assay with acetous perchloric acid in anhydrous acetic acid medium. Other methods based on fluorometric [8] and spectrophotometric [9–19] techniques have been reported for its assay in pharmaceuticals. Other than the official method [7], three more titrimetric methods are found in the literature for the assay of ETM in pharmaceuticals [20–22]. Reddy et al. [20] titrated ETM with N-bromosuccinamide using several anthraquinones as indicators. The drug in 25–500 *μ*mol levels was assayed by Ciesielski et al. [21] by titrating it with iodine in alkaline medium. Employing AgS-ion-selective electrode as the sensor, Obtemperanskaya et al. [22] have reported a micro method by titration of the drug solution with 0.01 M AgNO_3_. The titrant used in the previously reported method [20] is unstable and requires daily standardization whereas the method employing membrane electrode [22] is tedious and time-consuming. It is desirable that the methods used in routine analysis should be simple and rapid with minimum experimental operations. Though ETM is prone to oxidation, a stable and strong oxidant such as cerium (IV) did not figure among the several titrimetric or spectrophotometric reagents that have been employed earlier for the assay of ETM. The reported spectrophotometric methods suffer from some disadvantages such as need for longer contact time, pH adjustment, multistep reactions, extraction step, and dependence on critical experimental variables.

In this paper, we describe two simple, rapid, and sensitive methods for the determination of ETM in pharmaceuticals using cerium (IV) as the oxidant. The methods are based on the oxidation of ETM by a measured excess of cerium (IV) in H_2_SO_4_ medium followed by the determination of the unreacted oxidant either by titration with iron (II) visually (titrimetry) or by reacting it with ortho-dianisidine and measuring the absorbance of the orange-red coloured product at 470 nm (spectrophotometry). The two methods were found to be fairly accurate and precise in addition to being more sensitive compared to the previously reported methods.

## 2. Materials and Methods

### 2.1. Materials

Pharmaceutical grade ethionamide, certified to be 99.84% pure, was received as gift from Panacea Biotic Ltd. and used as received. Three brands of tablets, namely, Ethide (Lupin Ltd., Mumbai, India), Ethiokox (Radicura Private Ltd., New Delhi, India), and Myobid (Panacea Biotic, New Delhi, India) labeled to contain 250 mg of ETM per tablet, were purchased from local commercial sources.

### 2.2. Apparatus

A Systronics model 166 digital spectrophotometer (Systronics, Ahmedabad, Gujarat, India) with matched 1 cm quartz cells was used for absorbance measurements.

### 2.3. Chemicals and Reagents

All chemicals used were of analytical reagent grade. Double distilled water was used throughout the investigation.

*Cerium (IV) Solution (0.01 M).* An approximately 0.01 M cerium (IV) solution was prepared by dissolving the required quantity of cerium (IV) sulphate (Loba Chemie, Mumbai, India) in 0.5 M H_2_SO_4_ with the aid heat and filtered using glass wool; the solution was standardized [23] with pure ferrous ammonium sulphate (Loba Chemie, Mumbai, India) and used in titrimetry. The stock standard solution was diluted appropriately with 0.5 M H_2_SO_4_ to get 100 *μ*g/mL cerium (IV) for use in spectrophotometry.

*Ferrous Ammonium Sulphate, FAS (0.01 M).* It is prepared by dissolving the calculated amount of the chemical in water in the presence of few drops of dilute H_2_SO_4_ and standardized using pure potassium dichromate [23].

*Ortho-Dianisidine, ODS (0.05%).* It is prepared by dissolving the calculated amount of the chemical (Loba Chemie, Mumbai, India) in ethanol.

*Sulphuric Acid (5 M).* Concentrated acid (98%; sp. gr. 1.82, Merck, Mumbai, India) was diluted appropriately with water to get 5 M acid and used in spectrophotometry, and the same solution was diluted to 2 M level for use in titrimetry.

*Ferroin Indicator.* Prepared by dissolving 0.695 g of FeSO_4_·7H_2_O (Alpha Chemicals, India, assay 99%) and 1.485 g of 1,10-phenanthroline monohydrate (Qualigens Fine Chemicals, Mumbai, India, assay 100%) in water and diluted to volume in a 100 mL calibrated flask.

*Standard Drug Solution.* A solution of 1 mg/mL ETM was prepared by dissolving 250 mg of pure drug in 0.1 M H_2_SO_4_ and diluted to volume in a 250 mL calibration flask with the same solvent and used in titrimetric assay. The stock solution was diluted stepwise with 0.1 M H_2_SO_4_ to get a working concentration of 20 *μ*g/mL for spectrophotometry.

### 2.4. General Procedures

#### 2.4.1. Titrimetric Assay

A 10 mL aliquot of the drug solution containing 1.0–8.0 mg of ETM was placed in a 100 mL titration flask and acidified with 5 mL of 2 M H_2_SO_4_. Ten milliliters of 0.01 M cerium (IV) solution was pipetted into the flask and the contents were mixed well. After a standing time of 5 min, the residual oxidant was titrated with ferrous ammonium sulphate (FAS) solution using a drop of ferroin indicator. A blank titration was performed, and the amount in the aliquot was computed from the amount of cerium (IV) that reacted with ETM.

#### 2.4.2. Spectrophotometric Assay

Different aliquots (0.0,0.25,0.5,…, 2.5 mL) of 20 *μ*g/mL ETM solution were accurately transferred into a series of 10 mL calibrated flasks. To each flask 3 mL of 5 M H_2_SO_4_ was added, followed by 1 mL of 100 *μ*g/mL Ce(IV) solution. The contents were mixed well and the flasks were set aside for 10 min. Finally, 1 mL of 0.05% ODS solution was added to each flask, and the volume was brought to the mark with 5 M H_2_SO_4_. The absorbance of each solution was measured after 5 min at 470 nm against a water blank.

A standard graph was prepared by plotting the difference between blank absorbance and sample absorbance as a function of concentration of the drug, and the concentration of the unknown was computed using the regression equation derived from the absorbance-concentration data.

#### 2.4.3. Procedure for Tablets

Twenty tablets were weighed accurately and ground into a fine powder. A portion of the powder equivalent to 100 mg of ETM was weighed accurately and transferred into a 100 mL calibrated flask, 60 mL of 0.1 M H_2_SO_4_ was added, and the content was shaken for 20 min; the volume was diluted to the mark with 0.1 M H_2_SO_4_, mixed well, and filtered using Whatman 42 filter paper. The filtrate (1 mg/mL in ETM) was used in assay by titrimetry, and the same solution was diluted to 20 *μ*g/mL level for assay by spectrophotometry.

#### 2.4.4. Procedures for Method Validation

The assay validation procedures were carried out according to the current ICH guidelines [24], which include linear range, limits of detection (LOD) and quantification (LOQ), precision, accuracy, robustness, ruggedness, and selectivity.

*(1) Linear Range, LOD, and LOQ.* In titrimetry, the range was determined by titrating different amounts of drug under optimized conditions and the “*n*” value (number of moles of cerium (IV) reacting with each mole of ETM) was calculated. In spectrophotometry, the linearity was assessed by the calibration graph, which was constructed by plotting the absorbance versus concentration of ETM and the regression equation was calculated. The LOD and LOQ were calculated using the relation *ks*/*b* , where *k* = 3 for LOD and 10 for LOQ, *s* is the standard deviation of seven blank absorbance readings, and *b* is the slope of the calibration curve [25].

*(2) Accuracy and Precision.* The accuracy of the proposed methods was determined on the basis of the difference in mean calculated and amount/concentration taken (% deviation from the actual concentration, DFA); and the precision was determined by calculating the intraday and interday relative standard deviation. These were computed by analyzing standard solution of ETM at three levels seven times on the same day (intraday) and on five consecutive days (interday).

*(3) Robustness and Ruggedness.* Robustness was evaluated by assaying the standard solutions after slight but deliberate variations in the analytical conditions like contact time and volume of H_2_SO_4_. Ruggedness, on the other hand, was assessed by a study in which the determination was performed by three analysts and also by a single analyst using three different burettes (titrimetry) and cuvettes (spectrophotometry).

*(4) Selectivity.* The placebo blank and synthetic mixture were analyzed by the developed methods and the results compared with those obtained on standard drug solution. A placebo blank of the composition: 20 mg talc, 30 mg starch, 20 mg sucrose, 20 mg lactose, 10 mg gelatin, 20 mg sodium alginate, 30 mg magnesium stearate, and 20 mg methyl cellulose was prepared by homogeneous mixing in a mortar. Ten milligrams of placebo was placed in a 50 mL calibration flask and its extract was prepared as described under Section 2.4.3. To 50 mg of the placebo blank prepared above, 100 mg of pure ETM was added and mixed thoroughly and the mixture was quantitatively transferred into a 100 mL calibrated flask; and then steps described under Section 2.4.3 were followed.

*(5) Application to Tablets.* Tablet solution prepared as described earlier was subjected to analysis by applying the developed procedures by taking 5 mL aliquot (titrimetry) and 3 mL aliquot (spectrophotometry) in five replicates, and the measured analytical signal was used to calculate the percent of the label claim. For comparison, the tablet extract in glacial acetic acid was titrated potentiometrically with acetous perchloric acid [7].

*(6) Recovery Test.* Preanalyzed tablet powder was spiked with pure drug at three levels and the total quantity of the drug was calculated, and finally the percent recovery of the pure drug added was calculated.

## 3. Results and Discussion

Cerium (IV) sulphate is a chemical compound which is frequently used as an oxidizing agent in titrimetric methods. The orange colour of cerium (IV) ion is reduced to the colourless cerium (III) ion.

(1) $$\begin{array}{c} {Ce}^{+4}+e^{-}⇌{Ce}^{+3} \end{array}$$

Cerium (IV) is a powerful oxidizing agent which finds immense applications in the analysis of several pharmaceuticals [26–32]. This property of the oxidant was used in the present assay. The drug (ETM) was allowed to react with cerium (IV) in H_2_SO_4_ medium and gets oxidizing to its sulphoxide.
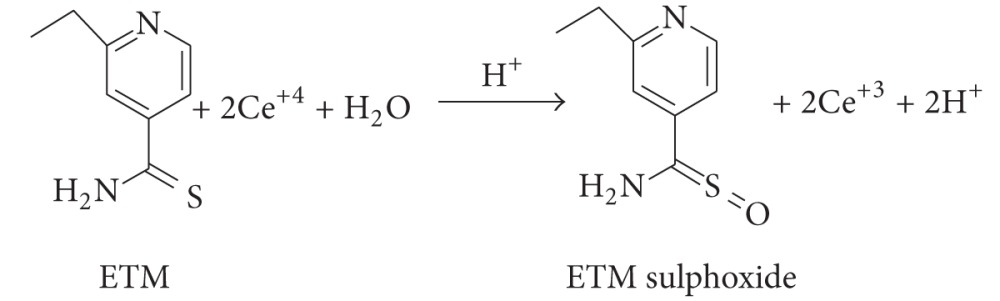
(2)


After an appropriate reaction time, the residual oxidant was determined by two approaches. In titrimetry, the unreacted oxidant was determined by titration with FAS using ferroin indicator.

(3) $$\begin{array}{c} {Ce}^{+4}+{Fe}^{+2}⇌{Ce}^{+3}+{Fe}^{+3} \end{array}$$

**Figure 1:**
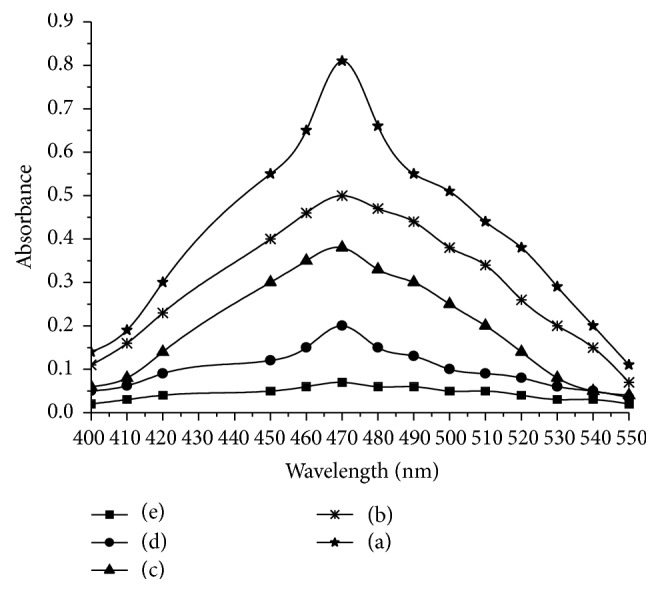
Absorption spectra of the reaction product in the presence of (a) 0.0; (b) 2.0; (c) 3.0; (d) 4.0; and (e) 5.0 *μ*g/mL ETM; the amount of other reactants remained constant.

The amount of cerium (IV) reacted was related to the amount of drug, and the drug-oxidant reaction followed a 1 : 2 stoichiometry which served as the basis of the calculations. In spectrophotometry, the unconsumed oxidant was determined by reacting with ODS as shown in (4) and measuring the absorbance of the coloured species of the oxidation product of ODS at 470 nm (Figure 1).
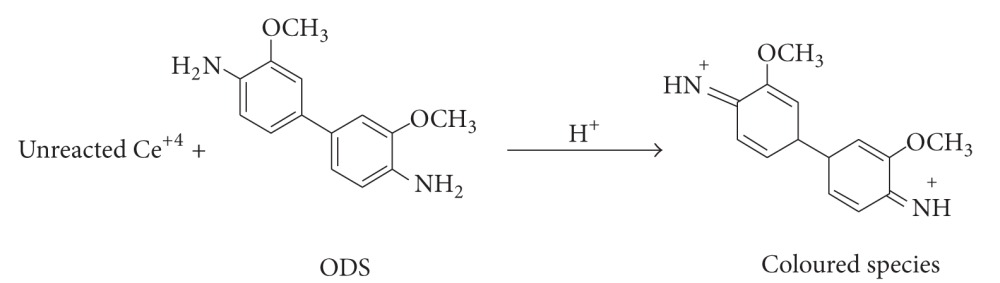
(4)


**Scheme 1:**
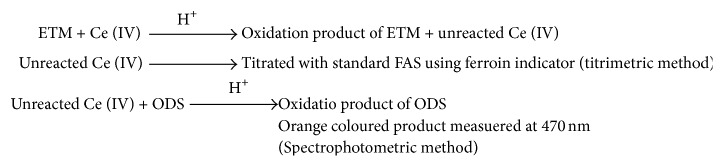
The possible reaction pathways and basis of assays.

**Figure 2:**
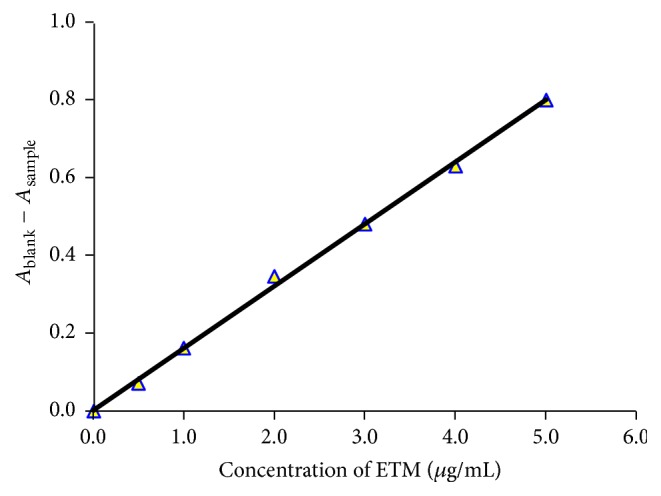
Calibration graph.

The calibration graph is a plot of the difference in absorbance of the reagent blank and sample solution versus the concentration of ETM (Figure 2), and this served as basis for the quantification. The possible reaction pathways and basis of assays are shown in Scheme 1.

In spectrophotometric method, three blanks were prepared. The first blank which contained all reactants except ETM gave maximum absorbance. The second blank contained only Ce (IV) and H_2_SO_4_. The third blank contained optimum amounts of ODS and acid. Since the last two blanks had negligible absorbance at 470 nm, measurements were made against double distilled water.

### 3.1. Method Development

Direct titration of ETM with cerium (IV) in different H_2_SO_4_ concentrations was not successful. Back titrimetric assay was possible in the presence of 5 mL of 2 M H_2_SO_4_ in a total volume of 25 mL (net, 0.4 M).

A contact time of 5 min was found optimum for the range (1–8 mg) studied with 0.01 M cerium (IV) solution. A fixed reaction stoichiometry of 1 : 2 (drug : oxidant) was found for the investigated range of ETM. Beyond these limits (<1 and >8 mg), slightly inconsistent reaction ratios were obtained.

The ability of cerium (IV) to oxidize ETM and also ODS to an orange-red coloured product was exploited for the indirect spectrophotometric assay. A slightly higher concentration of H_2_SO_4_ was required for the twin oxidation steps involved, and to stabilize the coloured product. The oxidation of drug took somewhat a longer time (10 min) as compared to titrimetry (5 min), and a further 5 min was required to stabilize the oxidation product of ODS, which was stable for the next 30 min thereafter.

### 3.2. Method Validation

#### 3.2.1. Linearity, LOD, and LOQ of Spectrophotometric Method

The absorbance-concentration plot was linear with a good correlation coefficient (0.9989) in the 0.5–5.0 *μ*g/mL range. Sensitivity parameters such as molar absorptivity (*ɛ*), Sandell's sensitivity, LOD, and LOQ along with the slope and intercept of the regression equation are compiled in Table 1. Low values of LOD and LOQ and high value of (*ɛ*) confirm the sensitivity of the method for the determination of ETM in bulk drug as well in drug product.

#### 3.2.2. Accuracy and Precision

Replicate determination of ETM in pure drug solution at three levels was performed on an intraday and interday basis as a part of the accuracy and precision evaluation of the proposed methods. Relative standard deviation (% RSD), a measure of precision, and relative error (% RE), an indicator of accuracy values, were calculated to be ≤2% (intraday) and <2.1% (interday), as shown in Table 2.

#### 3.2.3. Robustness and Ruggedness

To evaluate the robustness, two experimental variables, namely, contact time and acid concentration, were altered slightly deliberately, and the influence of these changes was studied on the performance of the methods. The performance remained unaffected as shown by small values of % RSD (≤2.31). Determination of drug in solution at three levels was done by using three different burettes in titrimetric method and three cuvettes in spectrophotometric method and also by three persons using the same equipment. The person-to-person and equipment-to-equipment variations did not significantly affect the results as shown in Table 3.

#### 3.2.4. Selectivity

To determine the selectivity of the described methods, placebo and synthetic mixture analyses were performed. Replicate analyses of placebo blank gave a titer value almost equal to that blank titration in titrimetry and absorbance value very much the same as the reagent blank in spectrophotometry. When the synthetic mixture was subjected to analysis, at three amount/concentration levels by the proposed methods, the percent recoveries of pure drug ranged from 99.34 ± 1.12 to 101.7 ± 2.34 indicating noninterference from the inactive ingredients.

#### 3.2.5. Application to Tablets

Three brands of tablets of 250 mg strength were analyzed by the proposed methods and the results are presented in Table 4. The same tablets were also analyzed by the reference method [7] for comparison. The results revealed that there is a close agreement between the results obtained by the proposed methods and those of the reference method, besides the label claim. When the results were statistically evaluated by applying Student's *t*-test for accuracy and variance ratio *F*-test for precision, the calculated *t*- and *F*-values did not exceed the tabulated values at the 95% confidence level and four degrees of freedom, suggesting that the proposed methods and the reference method have similar accuracy and precision.

#### 3.2.6. Accuracy by Recovery Study

Accuracy of the proposed methods was further confirmed by recovery study following the standard-addition procedure. The percent recovery values of pure drug added shown in Table 5 unambiguously demonstrate that inactive ingredients such as talc, gelatin, starch, magnesium stearate, sodium alginate, and methylcellulose do not interfere in the determination of the active ingredient.

## 4. Conclusions

The oxidation reaction between the ETM and cerium (IV) in acid medium was advantageously exploited for the development of two simple, rapid, cost-effective, and sensitive methods for the determination of ETM in pharmaceuticals. The methods use cheap and easily available chemicals and an inexpensive instrument which can be accessed in any industrial quality control laboratory. The methods employ a stable oxidant unlike the previously reported titrimetric and spectrophotometric methods. Titrimetry is applicable over a micro scale (<10 mg) compared to the reported titrimetric methods including the official method, which would require 300–500 mg per trial. The proposed spectrophotometric method has a molar absorptivity value of 2.66 × 10^4^ L/(mol·cm) with a linear dynamic range of 0.5–5.0 *μ*g/mL and is one of the most sensitive methods ever developed for ETM (Table 6). Hence, the proposed methods can be conveniently employed in laboratories which can ill-afford costly chromatographic techniques.

## Figures and Tables

**Table 1:** Sensitivity and regression parameters.

Parameter	Spectrophotometric method
*λ*_max_, nm	470
Colour stability	30 min
Linear range, *μ*g/mL	0.5–5.0
Molar absorptivity (*ε*), L/(mol·cm)	2.66 × 10^4^
Sandell sensitivity^*∗*^, *μ*g/cm^2^	0.0063
Limit of detection (LOD), *μ*g/mL	0.013
Limit of quantification (LOQ), *μ*g/mL	0.043
Regression equation, *Y* ^*∗∗*^	
Intercept (*a*)	0.0038
Slope (*b*)	0.1591
Standard deviation of *a* (*S*_*a*_)	9.89 × 10^−2^
Standard deviation of *b* (*S*_*b*_)	2.18 × 10^−2^
Regression coefficient (*r*)	0.9989

^*∗*^Limit of determination as the weight in *μ*g/mL of solution, which corresponds to an absorbance of *A* = 0.001 measured in a cuvette of cross-sectional area 1 cm^2^ and *l* = 1 cm. ^*∗∗*^ *Y* = *a* + *bX*, where *Y* is the absorbance, *X* is the concentration in *μ*g/mL, *a* is intercept, and *b* is slope.

**Table 2:** Evaluation of intraday and interday accuracy and precision.

Method	ETM taken (mg or *μ*g/mL)	Intraday accuracy and precision (*n* = 7)			Interday accuracy and precision (*n* = 5)		
		ETM found^a^ (mg or *μ*g/mL)	RSD^b^ %	RE^c^ %	ETM found (mg or *μ*g/mL)	RSD^b^ %	RE^c^ %
Titrimetry	2.0	2.01	1.12	0.50	1.97	1.74	1.50
	4.0	3.94	1.35	1.50	4.03	2.03	0.75
	6.0	6.08	1.55	1.33	6.11	1.69	1.83

Spectrophotometry	1.0	0.98	1.41	2.00	1.02	1.81	2.00
	2.0	1.97	1.38	1.50	2.03	1.57	1.50
	3.0	3.04	1.65	1.33	2.95	1.93	1.67

^a^Mean value of seven determinations; ^b^relative standard deviation (%); ^c^relative error (%).

**Table 3:** Method robustness and ruggedness expressed as intermediate precision (% RSD).

Method	ETM taken (mg or *μ*g/mL)	Robustness (% RSD)		Ruggedness (% RSD)	
		Parameters altered		Inter-analysts (*n* = 3)	Interburettes/cuvettes (*n* = 3)
		Contact time^*∗*^	Volume of H_2_SO_4_^*∗∗*^		
Titrimetry	2.0	0.88	0.67	1.33	1.91
	4.0	1.05	0.88	1.66	1.83
	6.0	1.24	1.58	1.11	2.11

Spectrophotometry	1.0	1.53	1.85	1.44	1.97
	2.0	2.31	1.17	0.97	1.73
	3.0	2.24	1.74	1.47	1.65

In titrimetry, ETM taken/found is in mg and the same was in *μ*g/mL in spectrophotometry. ^*∗*^Contact time used: 4, 5, and 6 min in titrimetric method; 8, 10, and 12 min in spectrophotometric method. ^*∗∗*^Volumes of H_2_SO_4_ were 4, 5, and 6 mL (2 M) in titrimetric method and 2.5, 3.0, and 3.5 mL (5 M) in spectrophotometric method.

**Table 4:** Results of analysis of tablets by the proposed methods and statistical comparison of the results with the reference method.

Tablet brand name	Nominal amount	Found^*∗*^ (% of nominal amount ± SD)		
		Reference method	Proposed methods	
			Titrimetry	Spectrophotometry
Ethide	250	100.2 ± 1.23	99.88 ± 1.02 *t* = 0.45 *F* = 1.45	100.44 ± 1.86 *t * ** = **0.24 *F* = 2.29

Ethiokox	250	96.22 ± 1.45	98.4 ± 1.71 *t * ** =** 2.17 *F* = 1.39	98.06 ± 2.46 *t * ** = **1.44 *F* = 2.88

Myobid	250	97.34 ± 1.54	98.96 ± 1.85 *t * ** =** 1.51 *F* = 1.44	95.84 ± 1.78 *t * ** = **1.43 *F* = 1.34

^*∗*^Mean value of five determinations.

(Tabulated *t*-value at the 95% confidence level and for four degrees of freedom is 2.78).

(Tabulated *F*-value at the 95% confidence level and for four degrees of freedom is 6.39).

**Table 5:** Results of recovery experiment through standard-addition method.

Method	Tablet studied	ETM in tablet (mg; *μ*g/mL)	Pure ETM added (mg; *μ*g/mL)	Total found (mg; *μ*g/mL)	Pure ETM recovered (percent ± SD^*∗*^)
Titrimetry	Ethide 250	2.99 2.99 2.99	1.5 3.0 4.5	4.55 5.92 7.48	101.43 ± 1.42 98.97 ± 0.84 100.11 ± 0.68

Spectrophotometry	Ethide 250	1.51 1.51 1.51	0.75 1.50 2.25	2.33 3.21 3.82	103.22 ± 2.44 104.82 ± 2.53 102.14 ± 2.35

^*∗*^Mean value of three determinations.

**Table:** (a) Spectrophotometry

SL number	Reagent/s	Methodology	Linear range (*µ*g/mL)	Remark	Ref.
1	DCNQ	Orange coloured product in ethanol measured at 440 nm	—	—	[9]
2	DCNQ	Red coloured product formed in the presence of ammonia in alcoholic medium measured at 540 nm	6–42	20 min contact time	[10]
3	Iron (III)	Purple-violet colour complex in acid medium measured at 510 nm	0–36	Less sensitive and the molar absorptivity is equal to 2.48 × 10^3^ L/(mol·cm)	[11]
4	Iron (III) PPD	Thionine compound measured at 600 nm	—	Multiple step reaction involved	[12]
5	Sodium nitroprusside	Orange coloured product in basic medium measured at 510 nm	—	—	[13]
6	Sodium nitroprusside	Orange-red complex measured at 490 nm	5–32	—	[14]
7	PAR-V^+5^	Ternary complex (1 : 1 : 1) extracted into chloroform and measured at 560 nm	0.2–20	30 min contact time, extraction step is required	[15]
8	Osmic acid	Light yellow coloured product formed at pH4 measured at 375 nm	0.25–40	60 min contact time, pH adjustment is required	[16]
9	NBS-CB	Unbleached dye colour measured in acid medium at 540 nm	0.2–5.0	Critical acid conc.; less stable reagent used	[17]
10	KMnO_4_	Blue coloured manganate in alkaline medium measured at 610 nm (direct method)	1–10	Critical NaOH conc. Reaction rate precariously dependent on experimental variables	[18]
		Absorbance at a fixed time of 20 min measured (kinetic method)	1–10		
11	Sodium azide-iodine	Decrease in absorbance at the 5th min measured at 348 nm (kinetic method)	10–100	Reaction rate precariously dependent on experimental conditions	[19]
*12*	*Ce(IV)/ODS*	*Orange-red coloured species measured at 470 nm*	*0.5–5.0*	*Nondrastic experimental conditions, no critical pH adjust, no heating or extraction step. Shorter contact time (15 min), no use of organic solvent*	*Present work*

**Table:** (b) Titrimetry

SL number	Reagent/s	End point detection	Linear range	Remark	Ref.
1	NBS	Visually	—	Less stable oxidant used	[20]
2	I_2_-NaOH	Potentiometrically	25–500 *μ* mol	Critically dependent on alkaline concentration	[21]
3	AgNO_3_	Potentiometrically	—	Preparation of AgS-sensor is tedious & cumbersome, expensive titrant used	[22]
*4*	*Ce(IV)/FAS*	*Unreacted Ce* ^4+^ * titrated *versus* FAS*	*1–8 mg*	*Stable titrant used, facile working conditions employed*	*Present work*

DCNQ: Dichloronaphthoquinone; PAR: 4-(2-pyridylazo) resorcinol; PPD: p-phenylenediamine; NBS: N-bromosuccinimide; CB: Celestine blue; ODS: ortho-dianisidine; FAS: ferrous ammonium sulphate.

## References

[1] Conte J. E., Golden J. A., Mcquitty M., Kipps J., Lin E. T., Zurlinden E. (2000). Effects of AIDS and gender on steady-state plasma and intrapulmonary ethionamide concentrations. *Antimicrobial Agents and Chemotherapy*.

[2] Ongaya V. A., Githui W. A., Meme H., Kiiyukia C., Juma E. (2012). High ethionamide resistance in Mycobacterium tuberculosis strains isolated in Kenya. *African Journal of Health Sciences*.

[3] Crofton J., Chaulet P., Maher D. (1997). *Guidelines for the Management of Multidrug-Resistant Tuberculosis*.

[4] Blanchard J. S. (1996). Molecular mechanisms of drug resistance in *Mycobacterium tuberculosis*. *Annual Review of Biochemistry*.

[5] Takayama K., Wang L., David H. L. (1972). Effect of isoniazid on the in vivo mycolic acid synthesis, cell growth, and viability of *Mycobacterium tuberculosis*. *Antimicrobial Agents and Chemotherapy*.

[6] Delgado J. N., Remers W. A. (1998). *Wilson and Gisvold's Textbook of Organic Medicinal and Pharmaceutical Chemistry*.

[7] The British Pharmacopeia (2009). *Volume I & II Monographs: Medicinal and Pharmaceutical Substances, Ethionamide*.

[8] Walash M. I., El-Brashy A. M., Metwally M. E.-S., Abdelal A. A. (2004). FLuorimetric determination of carbocisteine and ethionamide in drug formulation. *Acta Chimica Slovenica*.

[9] Bedair M. M. (1991). Use of 2,3-dichloro-1,4-naphthoquinone for the spectrophotometric assay of five thio compounds of pharmaceutical importance. *Alexandria Journal of Pharmaceutical Sciences*.

[10] Devani M. B., Shishoo C. J., Mody H. J., Raja P. K. (1974). Detection of thioamides: determination of ethionamide with 2,3-dichloro-1,4-naphthoquinone. *Journal of Pharmaceutical Sciences*.

[11] Shah A. K., Agrawal Y. K., Banerjee S. K. (1981). Spectrophotometric method for the rapid determination of microgram amounts of ethionamide. *Analytical Letters*.

[12] El-Din M. S., Belal F., Hassan S. (1988). Spectrophotometric determination of some pharmaceutically important thione-containing compounds. *Zentralblatt für Pharmazie, Pharmakotherapie und Laboratoriumsdiagnostik*.

[13] Ibrahim F. A. (1994). Colorimetric estimation of certain thione-compounds of pharmaceutical importance. *Mansoura Journal of Pharmaceutical Sciences*.

[14] Devani M. B., Shishoo C. J., Doshi K. (1981). A spectrophotometric determination of ethionamide in tablets. *Indian Journal of Pharmaceutical Sciences*.

[15] Sikorska-Tomicka H. (1993). Spectrophotometric determination of ethionamide and thionicotinamide with 4-(2-pyridylazo) resorcinol and vanadium. *Chemia Analityczna (Warsaw)*.

[16] Sikorska-Tomicka H. (1985). Spectrophotometric determination of ethionamide and thionicotinamide with osmic acid. *Mikrochimica Acta*.

[17] Sastry C. S. P., Srinivas K. R., Prasad K. M. M. K. (1996). Spectrophotometric determination of drugs in pharmaceutical formulations with N-bromosuccinimide and Celestine blue. *Mikrochimica Acta*.

[18] Walash M. I., El-Brashy A. M., Metwally M. S., Abdelal A. A. (2004). Spectrophotometric and kinetic determination of some sulphur containing drugs in bulk and drug formulations. *Bulletin of the Korean Chemical Society*.

[19] Walash M. I., Metwally M. E.-S., El-Brashy A. M., Abdelal A. A. (2003). Kinetic spectrophotometric determination of some sulfur containing compounds in pharmaceutical preparations and human serum. *Il Farmaco*.

[20] Reddy B. S., Krishna R. R., Sastry C. S. P. (1982). Titrimetric determination of some antitubercular drugs by sodium nitrite and N-bromosuccinimide (NBS) using internal indicators. *Indian Drugs*.

[21] Ciesielski W., Krenc A., Zlłobińska U. (2005). Potentiometric titration of thioamides and mercaptoacids with iodine in alkaline medium. *Chemia Analityczna*.

[22] Obtemperanskaya S. I., Buzlanova M. M., Karandi I. V., Shakhid R., Kashin A. N. (1996). Potentiometric determination of some drugs and other physiologically active substances using a silver sulfide ion-selective electrode. *Journal of Analytical Chemistry*.

[23] Vogel A. I. (1961). *A Textbook of Quantitative Inorganic Analysis*.

[24] ICH

[25] Miller J. C., Miller J. N. (1994). *Statistic for Analytical Chemistry*.

[26] Darwish I. A., Khedr A. S., Askal H. F., Mahmoud R. M. (2005). Simple fluorimetric method for determination of certain antiviral drugs via their oxidation with cerium (IV). *Il Farmaco*.

[27] Rajendraprasad N., Basavaiah K., Vinay K. B. (2011). Volumetric and spectrophotometric determination of oxcarbazepine in tablets. *Acta Chimica Slovenica*.

[28] Revanasiddappa H. D., Deepakumari H. N., Mallegowda S. M. (2011). Development and validation of indirect spectrophotometric methods for lamotrigine in pure and the tablet dosage forms. *Analele Universitatii din Bucuresti-Chimie*.

[29] Basavaiah K., Chandrashekar U., Prameela H. C. (2003). Cerimetric determination of propranolol in bulk drug form and in tablets. *Turkish Journal of Chemistry*.

[30] Raghu M. S., Basavaiah K., Prashanth K. N., Vinay K. B. (2013). Titrimetric and spectrophotometric methods for the assay of ketotifen using cerium(IV) and two reagents. *International Journal of Analytical Chemistry*.

[31] Askal H. F., Abdelmegeed O. H., Ali S. M. S., El-Hamd M. A. (2010). Spectrophotometric and spectrofluorimetric determination of 1,4-dihydropyridine drugs using potassium permanganate and cerium (IV) ammonium sulphate. *Bulletin of Pharmaceutical Sciences*.

[32] Basavaiah K., Devi O. Z., Tharpa K., Vinay K. B. (2008). Oxidimetric assay of simvastatin in pharmaceuticals using cerium (IV) and three dyes as reagents. *Proceedings of the Indian National Science Academy*.

